# Intrinsic vulnerability assessment of shallow aquifers of the sedimentary basin of southwestern Nigeria

**DOI:** 10.4102/jamba.v10i1.333

**Published:** 2018-03-29

**Authors:** Saheed A. Oke, Danie Vermeulen, Modreck Gomo

**Affiliations:** 1 Department of Civil Engineering, Central University of Technology, South Africa; 2 Institute for Groundwater Studies, University of Free State, South Africa

## Abstract

The shallow groundwater of the multi-layered sedimentary basin aquifer of southwestern Nigeria was assessed based on its intrinsic vulnerability property. The vulnerability evaluation involves determining the protective cover and infiltration condition of the unsaturated zone in the basin. This was achieved using the PI (P stands for protective cover effectiveness of the overlying lithology and I indicates the degree of infiltration bypass) vulnerability method of the European vulnerability approach. The PI method specifically measures the protection cover and the degree to which the protective cover is bypassed. Intrinsic parameters assessed were the subsoil, lithology, topsoil, recharge and fracturing for the protective cover. The saturated hydraulic conductivity of topsoil, infiltration processes and the lateral surface and subsurface flow were evaluated for the infiltration bypassed. The results show moderate to very low vulnerability areas. Low vulnerability areas were characterised by lithology with massive sandstone and limestone, subsoils of sandy loam texture, high slopes and high depth to water table. The moderate vulnerability areas were characterised by high rainfall and high recharge, low water table, unconsolidated sandstones and alluvium lithology. The intrinsic vulnerability properties shown in vulnerability maps will be a useful tool in planning and monitoring land use activities that can be of impact in groundwater pollution.

## Introduction

Aquifer vulnerability or susceptibility is a system property that refers to:

groundwater sensitivity to contamination and describes the relative tendency or likelihood for contaminants to reach a specified position in the groundwater system after introduction at some location above the uppermost aquifer. (National Research Council 1993:210)

Aquifer vulnerability is also seen as a means to synthesise complex hydrogeological information into a usable form by planners, decision- and policy-makers, geoscientists and the public (Liggett & Talwar 2009). The concept of groundwater vulnerability is based on the assumption that the physical environment provides some natural protection to groundwater against human impacts, especially with regard to contaminants entering the subsurface environment (Vrba & Zaporozec 1994). Groundwater vulnerability assessment represents a basis for protection zoning and land use planning, as it helps to find a balance between water protection on one hand and economic interests on the other hand.

Aquifer vulnerability maps are aimed mostly at giving a first general indication of the potential groundwater pollution risk to allow regulators, planners and developers to make better informed judgements on the proposed new developments and on priorities in groundwater quality protection and monitoring (Foster 1998). Therefore, the most important potential use of vulnerability maps is for aquifer protection and in sensitising public awareness which, in turn, may result in positive reactions or more informed land use decisions (Oke, 2017). Intrinsic vulnerability assessment do not consider the type or nature of contaminants but the geological, hydrological and hydrogeological properties of the earth material through which travelling contaminants can undergo processes such as retardation, degradation and filtration (Daly et al. 2002; Goldscheider 2003; Oke & Francoise 2017).

With many surface waters now polluted, the importance of groundwater as a source of drinking water has significantly increased in Nigeria (Adelana et al. 2008). Despite its importance, groundwater is often misused, usually poorly understood and rarely well-managed. The main threats to groundwater sustainability arise from the steady increase in demand for water (e.g. rising population and per capita use, increasing need for irrigation) and increased use and disposal of chemicals to the land surface. For instance, the estimated population of Nigeria increased from 88.9 million in 1991 to 160 million in 2012 (NPC 2014), whereas the estimated percentage depending on groundwater (shallow and deep aquifer) stood at 65.7% by 2013 (NPC 2014). A breakdown of this estimates shows that 36.3% rely on boreholes and 29.4% rely on hand dug wells (NPC 2014).

The fundamental concept of groundwater vulnerability is that some areas are more vulnerable to contamination than others (Oke & Fourie 2017; Vrba & Zaporozec 1994). The ultimate goal of a vulnerability map which this research aims to develop is the subdivision of the sedimentary areas under investigation into several units showing the different degrees of vulnerability or risk. Therefore, the primary aim of this work is to assess the intrinsic vulnerability conditions of shallow aquifer of the sedimentary basin using the PI method. This assessment will assist in prioritising groundwater protection measures and guide selection of the most vulnerable aquifers for further investigation, monitoring and protection. This is more important, considering that vulnerability assessment is gaining increasing attention in Nigeria on a regional sedimentary aquifers level after previous work of Edet (2004), Ojuri and Bankole (2013) and Oke et al. (2016).

## Study area and geological setting

**FIGURE 1:**
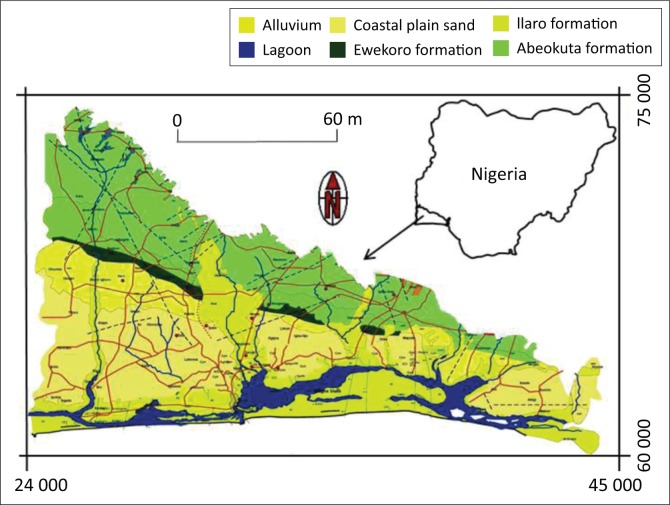
Location of sedimentary basin of southwestern Nigeria with respect to geology.

The study area is situated in the southwestern part of Nigeria (Figure 1). It is predominantly a sedimentary terrain with deposited beds lying conformably over one another. Poor land use practices, indiscriminate waste disposal and onsite sanitation systems in the form of septic tanks and pit latrines have caused contamination of groundwater resources in many urban areas in the basin especially where the groundwater table is shallow. The study area is a multi-layered aquifers system (Onwuka 1990). The stratigraphy of the basin consists of recent alluvium as the youngest hydrogeological formation and is underlain by the coastal plain sand (CPS) which consists of poorly sorted sands with lenses of claystones and mud. Thickness of almost 400 m was reported for the CPS towards the coast (Agagu 1985).

The CPS is the lateral equivalent of the petroleum rich Benin Formations of the Niger Delta Basin. The CPS is underlain by the whitish to yellowish, coarse sands of estuarine to marine Ilaro-Oshosun Formation. The Ilaro-Oshosun Formation is known for its high aquiferous potential. Groundwater abstraction range of 13.1 m^3^/h – 55.3 m^3^/h was reported (Offodile 2014). Its mineralogical composition is characterised by poorly sorted quartz, greenish-grey or beige clay, white to purple clay and unconsolidated clayey shale. The Abeokuta Formation is the oldest sedimentary deposition in the basin (Kogbe 1976). It unconformably overlies the Basement Complex rocks of southwestern Nigeria. The formation consists mainly of poorly sorted ferruginous grits, siltstones and mudstones.

## Methodology

Groundwater vulnerability mapping has advanced since the use of the term aquifer vulnerability by Margat (1968). Ever since then, vulnerability methods have improved considerably. One of the best recently developed and widely used intrinsic vulnerability method which is applicable to most karst and non-karst area is the PI method. The method was developed by Goldscheider et al. (2002, 2003) within the scope of COST Action 620 Project ‘Vulnerability and Risk Mapping for the protection of Karst Aquifers’ and it forms part of the European Approach to vulnerability mapping (Daly et al. 2002).

The PI method was adopted to assess the intrinsic vulnerability of the sedimentary basin under investigation because of the high number of input parameters in its methodology and its design for resources assessment. This has been attested to by Neukum, Hötzl and Himmelsbach (2008) and Ravbar and Goldscheider (2009). The PI method has been further developed to include source and resources vulnerability assessment by Ravbar and Goldscheider (2007). The resources assessment targets groundwater table and pathways through the layers above the water table, while the source assessment targets groundwater in wells, springs and horizontal movement within the aquifer as pathways. However, it should be noted that it is possible to protect source without protecting the resources.

The P acronym stands for protective cover effectiveness of the overlying lithology and I stands for the degree of infiltration bypass. The protective cover (P) is a modified German vulnerability method (GLA) proposed by the German State Geological Survey (Holting et al. 1995). The P-factor is based upon the concept of natural lithological and sediment infiltration and attenuation capacity. This is measured with the amount of input parameters described in equation 1. The final protective function (*P*_TS_) is calculated using the formula in equation 1:

$$P_{TS}=\left[T+\left(\sum_{i=1}^{m}S_{i}M_{i}+\sum_{i=1}^{n}B_{i}M_{i}\right)\right]R+A$$ [Eqn 1]

where *T* refers to topsoil (up to 1 m), *S* = subsoil, *B* = bedrock, *M* is the thickness of each layer in metre, *R* is the recharge factor, *A* is the artesian pressure factor at the potentiometric surface level, *m* is number of subsoil layers and *n* is the bedrock layers. The factor *B* presents the product of *B* = *LF*, where *L* depends on the type of bedrock and *F* on the degree of fracturing or karstification. The P-factor is scaled according to Table 1.

**TABLE 1:** Rating of the P-factor according to Goldscheider (2003).

Score P_TS_	Effectiveness of protective cover	P-factor	Example
0–10	Very low	1	0 m – 2 m gravel
> 10–100	Low	2	1 m – 10 m sand and gravel
> 100–1000	Medium	3	2 m – 20 m slightly silty sand
> 1000–10 000	High	4	2 m – 20 m clay
> 100 000	Very high	5	> 20 m clay

*Source*: Goldscheider, N., 2002, ‘Hydrogeology and vulnerability of karst systems, examples from the Northern Alps and Swabian Alb’, PhD thesis, University of Karlsruhe

**FIGURE 2:**
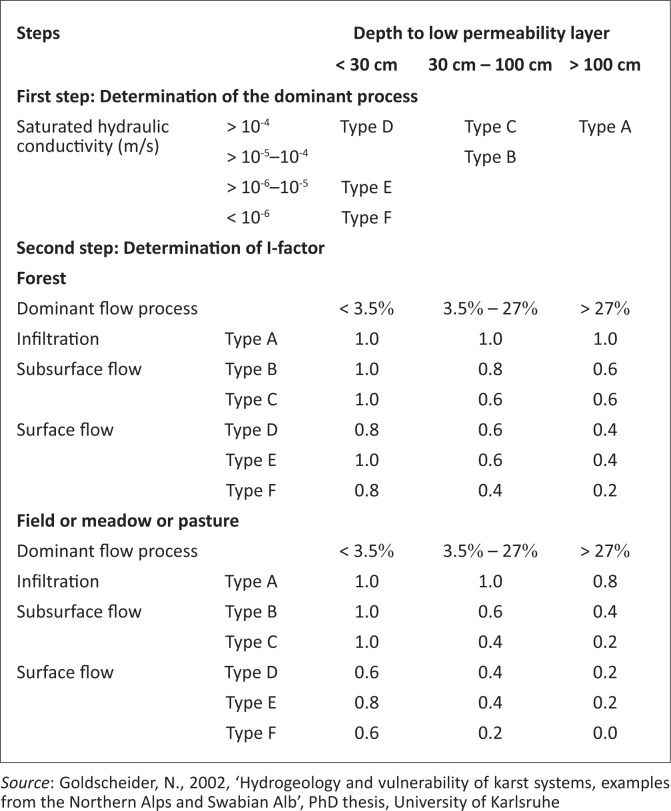
Step for the determination of dominant flow as presented by Goldscheider (2000).

The I-factor describes the infiltration conditions, particularly the degree to which the protective cover is bypassed as a result of lateral surface and subsurface flow. Therefore, the I-factor distinguishes between the dominant flow processes (infiltration, subsurface flow and surface flow). These processes are determined for the dominant flow in karst areas specifically. However, in situ geology in the Dahomey Basin is not karstic, but I-factor was used to assess the infiltration conditions exclusively for the saturated hydraulic conductivity of the top and subsoils. The rating of the flow processes as presented by Goldscheider (2002) is presented in Figure 2. The vulnerability map was prepared with Windows Systems Software for Hydrogeologist (WISH). This software is compatible with most GIS mapping software.

## Results

### The P-map

The P-map includes assessment of the protective cover over the shallow unconfined aquifer. It practically assesses the vadose zone properties. The vadose zone thickness decreases towards the coastal areas. The P-map parameters assessed covered a wide range of rocks. The input parameters of protective cover are as follows:

- effective field capacity (eFC) of the topsoil up to 1 m depth
- infiltration
- type of the subsoil
- type of the bedrock and degree of its fracturing
- thickness of each layer above the groundwater table
- presence or lack of permanent artesian conditions.

### Topsoil (T)

The topsoil is represented by the eFC. The eFC is defined as the portion of the field capacity which is available to plants in certain soil types. The eFC was termed available water capacity (AWC) in the GLA method upon which the PI was based. Typical values of soil texture as presented by Hennings (2012) include 10 mm/dm for (sandy loam), 10.5 mm/dm for sandy clay loam, 8.5 mm/dm for sandy clay and 7.5 mm/dm for clay. The dm (decimetre) represents the estimated depth of soil profile. The major compositions of the sedimentary basin topsoils are sands and some layers of sandy loam. Goldscheider (2005) reported eFC on gravel and sand of the Engen Test Site in Germany and reported a low to medium eFC (50 m – 140 m). Nick (2011) suggested to choose the field capacity based on textural classification. Considering that topsoils in the recent alluvium and coastal plain sand are loose, porous sand and PI method do not explicitly cater for unconsolidated sediments. Therefore, the effect of effective water capacity up to 1 m depth is low, and was rated 0–50 as presented in Table 2, whereas the other geological formations were rated higher, that is, > 50 mm – 90 mm. This consists of shale, bedded sandstone and lateritic sediments.

**TABLE 2:** Effective water capacity for topsoil classification.

eFc (mm) up to 1 m depth	*T*
> 250	750
200–250	500
140–200	250
90–140	125
50–90	50
< 50	0

*Source*: Goldscheider, N., 2002, ‘Hydrogeology and vulnerability of karst systems, examples from the Northern Alps and Swabian Alb’, PhD thesis, University of Karlsruhe

eFc, effective field capacity; T, Topsoil

### Recharge (R)

The factor R is assessed based on the value of the groundwater recharge. The recharge estimation in the PI method is similar to the recharge of other vulnerability methods. Xu and Braune (2010) reported a recharge rate > 500 mm/y for southern Nigeria including the sedimentary basin of the southwestern areas. This is partly because of the high rainfall experience in most areas of the basin. Average annual precipitation in the Lagos part of the basin records amount around 1800 mm/y and the lowest annual precipitation of 1200 mm/y in Abeokuta areas (Oke et al. 2013). The PI rating of recharge is shown in Table 3. The basin’s spatial recharge shows recharge values above 400 mm/y which is 0.75.

**TABLE 3:** Recharge rating of the PI protective cover.

Recharge (mm/y)	*R*
0–100	1.75
> 100–200	1.50
> 200–300	1.25
> 300–400	1.00
> 400	0.75

*Source*: Goldscheider, N., 2002, ‘Hydrogeology and vulnerability of karst systems, examples from the Northern Alps and Swabian Alb’, PhD thesis, University of Karlsruhe

R, recharge

### Subsoil (S)

The subsoil in the PI method is defined as the soil interval beyond 1 m from the surface (Goldscheider et al. 2000). In the basin, soil profile is as thick as the vadose zone in most places with values from 3 m to 45 m range. This is because of the sediment compositions of depositional material. The thick soil profiles were enhanced by active weathering which results from the seasonality of the weather and tropical climatic belt, with temperature above 30 °C per day. The type of subsoil and textural class depends on their grain size distribution (GSD). A range used in the subsoil classification in calculating the protective cover is presented in Table 4. The subsoil thickness of 5 was used in the recent alluvium predominant southern areas for the vadose thickness, whereas sand subsoil value was 25 and sandy loam was 180.

**TABLE 4:** Subsoil range in P-factor.

Type of subsoil base on grain size distribution	S
Silty loam	220
Sandy silty loam, slightly sandy loam	200
Sandy loam	180
Slightly silty sand	50
Sand	25
Sand with gravel, sandy gravel	10

*Source*: Goldscheider, N., 2002, ‘Hydrogeology and vulnerability of karst systems, examples from the Northern Alps and Swabian Alb’, PhD thesis, University of Karlsruhe

S, subsoil

### Lithology (L)

**FIGURE 3:**
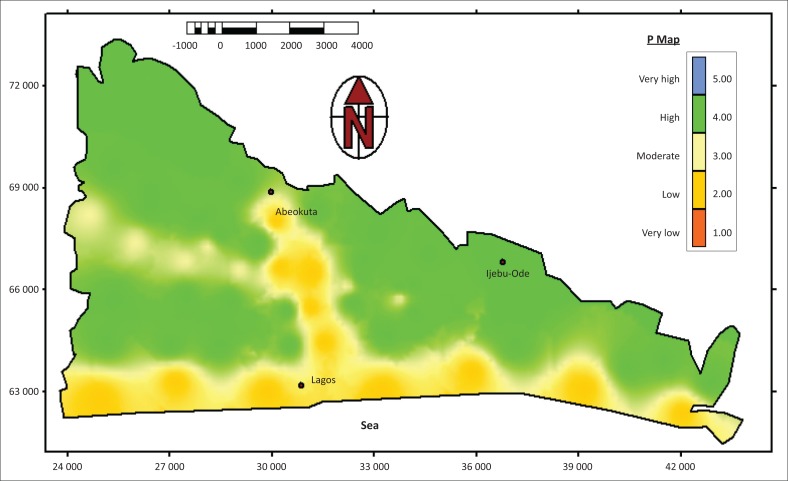
Protective cover map of sedimentary basin of southwestern Nigeria.

The geological map of Nigeria guided the lithology classification. Sandstones (porous sandstone, massive sandstone and bedded sandstone), alluvial deposits and limestone are the major rock types dominating the sedimentary basin. Table 5 shows the lithology and values assigned to the rock types. The lithologies used were strictly those above the water table and below the ground surface. Depth to water table measured were 3.0 m – 4.5 m along the coastal areas, 7 m – 20 m at Central Lagos and Shagamu, 45 m at Ijebu Ode areas and 15 m – 30 m at Abeokuta areas. This means that other rock types present in the basin below these water tables were not considered. This is because the groundwater table are usually the targeted point in groundwater protection in resources vulnerability studies (Goldshcheider 2002; Zwahlen 2003). Ratings were based on the presented lithology in Table 5. Fracturing were considered to be absent or with few occurrences in the basin because of high impact of weathering as witnessed in most tropical regions of the world and assigned a value of one. In addition, artesian pressure was not considered in this study because the shallow unconfined aquifer systems were targeted with no artesian pressure. The final protective cover vulnerability map is presented in Figure 3. The P-map ranged from low (2) to high class (4). This means the protective cover of the sedimentary basin is slightly effective.

**TABLE 5:** Values of lithology in the P-factors.

Lithology	*L*
Marl, siltstone, claystone	20
Sandstone, quartzite, metamorphite	15
Porous sandstone, tuff	10
Limestone, conglomerate	5

*Source*: Goldscheider, N., 2002, ‘Hydrogeology and vulnerability of karst systems, examples from the Northern Alps and Swabian Alb’, PhD thesis, University of Karlsruhe

L, lithology

### The I-map

The I-factor shows the degree to which the protective cover is bypassed by lateral surface and subsurface flow and subsequent concentrated recharge. The following 3 steps as stated by Goldscheider (2002) were considered in order to determine the I-factor and I-map construction, respectively:

- saturated hydraulic conductivity of topsoil
- infiltration processes
- lateral surface and subsurface flow.

**FIGURE 4:**
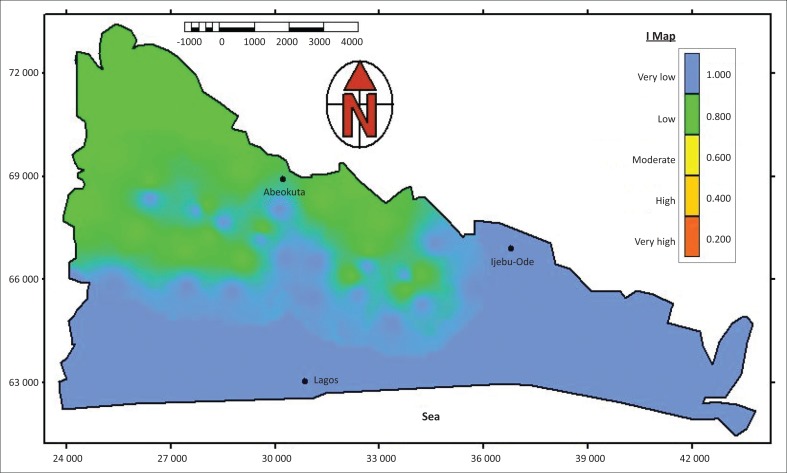
The I-map of the sedimentary basin.

The topsoil properties decide the dominant flow process. The basin falls within type A for the dominant flow process (Figure 2). Infiltration flow predominates on high permeability soils (> 100 cm) and saturated hydraulic conductivity (> 10^−4^ m/s) with type A as was the case in the basin. Infiltration flow and subsequent percolation takes place in permeable topsoils overlying layers with low permeability layers. Infiltration processes and run-off generation are also influenced by the slope gradient and vegetation. Gentle slopes and forests (natural forests and plantations) favour infiltration and percolation. Steep slopes and agricultural land favour run-off. Northern areas of the sedimentary basin contain steep slopes and thick vegetation and are assigned a value of 0.8 (Figure 2), whereas the southern end is relatively flat and contains swampy vegetation and a value of 1 is assigned (Figure 2). Consequently, the I-map (showing the degree to which the protective cover is bypassed) is obtained by intersecting the I-map (showing the occurrence and intensity of lateral flow) with the surface catchment map by using a GIS raster calculation. This is shown in Figure 4.

### Vulnerability map

**FIGURE 5:**
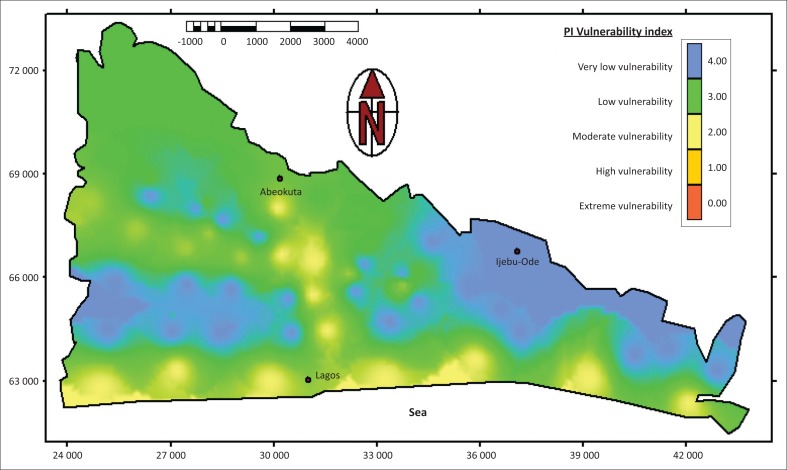
PI vulnerability map of the Dahomey Basin.

The final vulnerability map is the product of the P-map and I-map and is presented in Figure 5. As shown in the map, the vulnerability class of the sedimentary basin ranges from moderate to very low vulnerability with PI vulnerability index of range of 2–4. The PI method rated its classes with specified colours, namely very high vulnerability (red, 0–1), high (orange, 1–2), moderate vulnerability (yellow, 2–3), low vulnerability (green, 3–4) and very low vulnerability (blue, 4–5). This is shown in Table 6. The index of vulnerability (Π-factor) was evaluated based on P- and I-factors. Low protective function of overlying layers result in high vulnerability and vice versa. In general, 46% of the basin is classified as very low vulnerability, 20*%* as low vulnerability and 34% as moderate vulnerability.

**TABLE 6:** Index of vulnerability map derived from P-factor, I-factor and PI vulnerability map.

Colour	Vulnerability map Vulnerability of groundwater		P-map Protection function of overlying layers		I-map Degree of bypassing	
	Description	II-factor	Description	P-factor	Description	I-factor
Red	Extreme	> 0–1	Very low	1	Very high	0–0.2
Orange	High	> 1–2	Low	2	High	0.4
Yellow	Moderate	> 2–3	Moderate	3	Moderate	0.6
Green	Low	> 3–4	High	4	Low	0.8
Blue	Very low	> 4–5	Very high	5	Very low	1.0

*Source*: Goldscheider, N., 2002, ‘Hydrogeology and vulnerability of karst systems, examples from the Northern Alps and Swabian Alb’, PhD thesis, University of Karlsruhe

Areas showing moderate vulnerability is characterised by low thickness of subsoil, high rainfall (1800 mm/y) and a very low water table (< 5 m), and it covers areas of unconsolidated alluvium and porous sandstone sediments. Low vulnerability areas result from lithology containing sandstone and sandy loam soil texture. Slopes are high (> 10 m above the sea level), saturated hydraulic conductivity of the topsoil supports infiltration and the water table is relatively high (> 30 m). Very low vulnerability areas that are represented with the blue colour are massive sandstone with loose topsoils. The thickness of the lithology and overall depth to the water table is high (15 m – 21 m). However, the very low vulnerability areas contain flat topography that supports high infiltration, but because of the rating of other factors (particularly the alluvium sediment with appreciable clay content), it shows little significance in the overall vulnerability map. It is expected that flat topography encourages ponding, gradual infiltration and subsequent percolation. Vulnerability assessment requires quality assurance or validation of vulnerability maps, which are based on data not used in the vulnerability assessment method.

**FIGURE 6:**
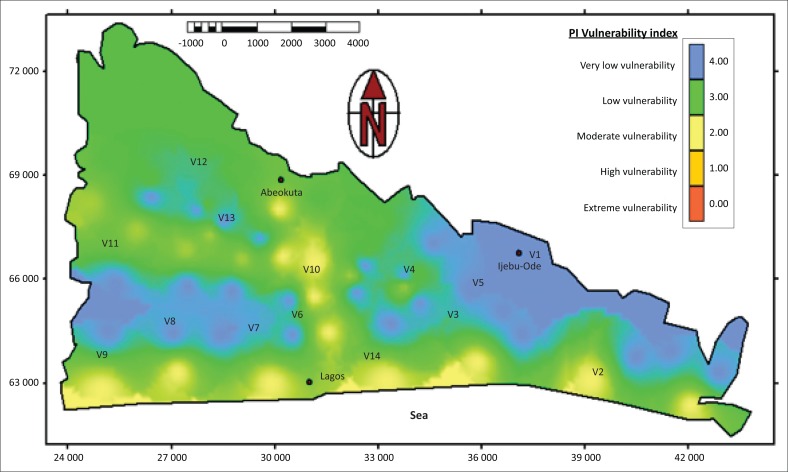
Validation map showing groundwater monitoring points.

Validation techniques in vulnerability assessments include using hydrographs, bacteriology, tracer techniques, extensive monitored chemical property and many others (Daly et al. 2002). The techniques used in validating the PI vulnerability map involved monitoring of the chemical properties of selected elements in selected locations of the basin. These include anions (NO_3_ and Cl), cations (Na and Ca) and trace elements of Cu and Zn (Table 7). The elements were selected based on their environmental signatures such as conservative chloride that gives recharge sources, nitrate, zinc and copper that show traces of anthropogenic sources in the groundwater and sodium and calcium that show water–rock interactions properties and sea influences. Figure 6 shows the 14 selected hydrochemical sampling points cutting across the vulnerability classes. These are V2, V10 and V14 (moderate vulnerability); V11, V12, V9, V4 and V6 (low vulnerability); and V1, V3, V5, V8, V7 and V13 (very low vulnerability).

**TABLE 7:** Hydrochemical results of selected locations in the basin.

Field number	Locations	Na	Ca	NO_3_	Cl	Cu	Zn
V1	Ijebu ode	7.88	12.37	0.10	90	0.029	0.114
V2	Epe	15.92	2.57	1.35	144	0.026	0.029
V3	Odelemo	19.61	12.60	0.10	90	0.024	0.040
V4	Shagamu	12.54	4.79	0.10	108	0.029	0.119
V5	Aiyepe	6.67	8.89	0.10	90	0.046	0.044
V6	Akute-Ijoko	6.24	6.24	1.81	126	0.102	0.205
V7	Sango-Ota	32.71	4.51	0.70	108	0.047	0.035
V8	Ado Odo	10.21	3.21	0.10	72	0.042	0.033
V9	Ipokia	9.29	2.13	0.10	108	0.052	0.043
V10	Papalanto	117.51	59.43	0.10	90	0.031	0.038
V11	Oja Odan	37.68	23.67	0.10	90	0.046	0.038
V12	Ayetoro	11.52	9.77	0.10	90	0.034	0.044
V13	Iboro	7.65	0.85	1.69	144	0.031	0.040
V14	Ikorodu	52.23	57.72	1.34	108	0.046	0.050
WHO (2011)	-	200.00	75.00	50.00	250	2.000	3.000
SANS 241 (2015)	-	200.00	150.00–300.00	11.00	300	2.000	5.000

All samples are in Mg/L.

Na, Sodium; Ca, Calcium; NO_3_-, nitrate ion; Cl, Chloride; Cu, Copper; Zn, Zinc; WHO, World Health Organisation; SANS, South African National Drinking Water Standard.

The hydrochemical results were compared with the drinking water standard of World Health Organization (WHO 2011) and South African National Standard (SANS 2015). The validation samples were within acceptable drinking standards (Table 7). Nitrate, copper and zinc were insignificant in all the samples. Samples from the moderate vulnerability areas were higher than the low and very low vulnerability areas. A major intrinsic vulnerability parameter responsible for the higher concentration of elements is the low depth to water in the moderate vulnerability areas compared to the other assessed areas. At V10 (Papalanto junction), unconfined aquifer water table at 3 m below ground level overlies the shale and clay deposits of Akinbo/Oshosun Formations, whereas at V14 (Ikorodu) and V2 (Epe), the shallow groundwater level varies between 6 m and 8 m below ground level.

## Discussions

The PI vulnerability method assessed in this study was based on important intrinsic parameters that can evaluate the risk of groundwater to contamination. The most important intrinsic vulnerability property of groundwater risk to contamination is depth to water, lithology, recharge and infiltration. The sedimentary basin aquifers with moderate vulnerability areas should be monitored closely, whereas areas with low vulnerability may not require detailed monitoring. Furthermore, strict monitoring and permissions or consents for environmental activities should have more demanding conditions imposed on areas of moderate as opposed to very low vulnerability.

The vulnerability map of the sedimentary basin produced here is based on the available information at the time of production and will require periodic updating. Special warnings to the use of vulnerability maps are as follows:

- All groundwater is to some degree vulnerable.
- Uncertainty is inherent in all vulnerability assessments.
- There is risk that the obvious may be obscured and the subtle indistinguishable (NRC 1993).

It is also recommended that land use practice of the basin’s most vulnerable areas should be monitored and inappropriate land use should be stopped to avoid pollutants entering the shallow groundwater. This is resources vulnerability assessment and not source assessment. Therefore, it is not applicable to well or spring protection.

## Conclusion

Groundwater vulnerability assessments carried out in this article are based on the geological intrinsic properties of the overlying lithology above the unconfined aquifer systems of the sedimentary basin of southwestern Nigeria. The vulnerability maps were derived from the PI methodology which is based on the European concept of aquifer vulnerability assessments. Intrinsic assessment of the sedimentary basin shows degree of vulnerability ranging from moderate to very low. The protective cover of lithology above the unconfined aquifer of the sedimentary basin ranges from low to high covers. This has shown that the basin is not entirely protected thereby suggesting need for prioritising high groundwater vulnerability zones. Presently, the groundwater protection law in Nigeria is inadequate, and land use conditions are practically unmonitored. This research has shown areas where priority protection zoning should be concentrated from land use activities that may be of high risk to the shallow groundwater of the basin.
